# Hypocalciuric Hypercalcemia due to Impaired Renal Tubular Calcium Excretion in a Type 2 Diabetic Patient

**DOI:** 10.1155/2017/3694868

**Published:** 2017-04-11

**Authors:** Sihao Yang, Yan Ren, Xi Li, Haoming Tian, Zhenmei An, Tao Chen

**Affiliations:** ^1^Department of Endocrinology and Metabolism, West China Hospital of Sichuan University, Chengdu, China; ^2^Department of Chinese Traditional Medicine, The Second People's Hospital of Yibin, Yibin, China

## Abstract

The case we presented here was a 73-year-old gentleman, who was admitted to endocrinology department due to recurrent fatigue for 1 year. He had medical histories of type 2 diabetes for 18 years and developed CKD 4 years ago. He also suffered from dilated cardiomyopathy, and coronary heart disease, moderate sleep apnea syndrome, primary hypothyroidism, and gout. His treatment regimen was complicated which included Caltrate D and compound *α*-keto acid (1200 mg calcium/d). Laboratory examination revealed that his serum calcium level elevated, 24-hour urine calcium output decreased, PTH level was suppressed, and 25-hydroxyvitamin D was in normal low range. No other specific abnormalities were found in serum bone turnover markers, ultrasonography, computed tomography, and bone scintigraphy. The diagnosis was suggested to be hypocalciuric hypercalcemia but was different from familial or acquired hypocalciuric hypercalcemia which were featured by elevated PTH level. The patient was asked to restrict calcium intake and to take diuretics; then his serum calcium level gradually lowered. In brief, patients with CKD could present with hypocalciuric hypercalcemia due to impaired renal calcium excretion. In this case, calcium restriction should be applied for treatment.

## 1. Introduction

Hypercalcemia related to PTH, malignancy diseases (PTHrP or destruction), and vitamin D metabolites usually could be easily diagnosed according to clinical setting [1]. However, in other cases, especially in cases with PTH independent hypercalcemia, the etiology is often difficult to identify. A published review summarized the rare causes of hypercalcemia and showed the potential mechanisms including calcitriol overdosage, occult milk-alkali syndrome, some medications (e.g., omeprazole, theophylline toxicity, and growth hormone), and other diversity causes [2]. Other recent studies showed that sarcoidosis [3], granulomatosis/granuloma [4, 5], diabetic ketoacidosis [6], and methylmethacrylate for cosmetic purposes [7] could also be causes for PTH independent hypercalcemia. However, hypercalcemia due to impaired renal calcium excretion was rarely reported. Here, we presented a case of hypocalciuria hypercalcemia with suppressed PTH levels. The mechanism could be impaired renal tubular calcium regulation.

## 2. Case Report

The patient was a 73-year-old man who was admitted in September 21, 2015, due to recurrent fatigue for 1 year. He was diagnosed as type 2 diabetes 18 years ago, developed chronic kidney disease 4 years ago, and began to take compound *α*-keto acid (2520 mg three times a day, approximately containing 600 mg calcium element). Six years ago, he was diagnosed with osteoporosis and began to take Caltrate D (600 mg calcium element) and Alphacalcidol (0.5 *μ*g) daily. One year ago, the patient began to feel fatigue. Laboratory tests showed serum calcium elevated to 3.41 mmol/L. After stopping Caltrate D and Alphacalcidol, it fluctuated between 2.24 and 2.93 mmol/L. Two months ago, he felt fatigue again and was referred to endocrinology department. Laboratory tests showed elevated serum calcium level (3.03 mmol). No other clinical significant changes were found in thyroid function, blood gas analysis, immunologic test, protein electrophoresis, tumor markers, ACTH, and plasma total cortisol levels.

We then comprehensively reviewed his laboratory reports in recent years (Table 1). We found his serum calcium began to raise at the end of 2013, as indicated by suppressed PTH (serum calcium 2.67 mmol/L, PTH 0.76 pmol/L), and then raised up to 3.41 mmol/L in 2014. After calcium and vitamin D restriction, his serum calcium fluctuated between 2.24 and 2.93 mmol/L, and PTH were consistently suppressed (from 0.79 to 0.82 pmol/L). Further evaluation showed that his urine calcium output decreased (1.63–2.51, reference value 2.5–7.5 mmol/24 hours) when serum calcium increased. Meanwhile, his serum 25-hydroxyvitamin D, bALP, and CTX were all in low levels. No specific signs were found by DEXA, chest and abdominal computed tomography, and bone scintigraphy.

Taken together, we suggested that this patient developed a clinical condition, featured by hypocalciuric hypercalcemia and suppressed PTH level. He was asked to cease compound *α*-keto acid, restrict milk intake, increase water intake, and use diuretics. His serum calcium gradually returned to normal. On March 16, 2016, he experienced recurrent cramp in both legs. Laboratory workup showed his serum calcium decreased to 2.02 mmol/L, and PTH elevated to 11.44 nmol/L. His treatment was adjusted to compound *α*-keto acid two tablets one day, three times one week, and he was asked to monitor serum calcium every month.

## 3. Discussion

Hypocalciuric hypercalcemia as familial pattern was well-known [1]. In recent two decades, acquired hypocalciuric hypercalcemia was reported and was supposed to be resulting from autoantibody against calcium-sensing receptor (CSRP) [8, 9]. A typical feature of acquired hypocalciuric hypercalcemia slightly elevated PTH level, which is due to increased set point of parathyroid cell for serum calcium. While, in this case, PTH was suppressed when serum calcium increased, and PTH elevated when serum calcium decreased, indicating normal function of CSRP in this patient's parathyroid gland. Thus, we deduced that his hypercalcemia was caused by impaired calcium excretion from urine.

In normal person, about 59% of total serum calcium is filtered into crude urine. Then, 90% of those were reabsorbed by proximal tubules, henry loop, and early distal tubules and nearly 10% reabsorbed by early collecting ducts and late distal tubular. The latter portion is relatively few but more important in calcium hemostasis because it is highly dependent on the blood calcium ion concentration. A slightly elevation of serum calcium level, under the regulation of PTH, will result in strikingly increasing calcium excretion from urine [8, 10].

In this case, the patient's serum PTH level changed corresponding to alteration of serum calcium level but failed to maintain serum calcium to the normal range. Thus, we proposed that the patient's hypocalciuric hypocalcemia resulted from impaired response of late distal tubular and early collecting ducts to the changed PTH level. We searched Medline (PubMed, update to May 2, 2016) using Mesh term “Hypocalciuric Hypercalcemia” but failed to retrieve any similar reports. We were unable to perform renal biopsy because of the patient's refusal. So, we could not, regrettably, demonstrate the exact mechanism of how the patient's renal tubular failed to respond to changed serum calcium level. The differential diagnosis of hypercalcemia without clear etiology is often very difficult; therefore, the case we presented here might provide some valuable information for future clinical practice.

## 4. Conclusion

PTH independent hypocalcaemia could be caused by decreased urine calcium excretion in patients with complicated clinical conditions. Calcium restriction and carefully monitoring were the key points in treatment.

## Figures and Tables

**Table 1:** The changes of the patient's serum and urine calcium levels and their related biochemical indexes in recent 7 years.

	2009-10-28	2011-12-16	2012-1 to 2013-11	2013-12-14	2014-10-25	2014-11 to 2015-6	**2015-09-21**	2016-3-16	2016-4 to 2016-8
Events	Calcium 600 mg/d, Alphacalcidol 50 *μ*g/d	Added compound *α*-keto acid (600 mg calcium/d)		Initially suspected hypercalcemia during review	Diagnosed as hypercalcemia; ceased calcium, Alphacalcidol		This admission; ceased compound *α*-keto acid		Compound *α*-keto acid (150 mg calcium/d)
Serum BUN	7.15	17.62	9.2–12.1	11.66	11.78	13.36–20.65	15.8	22.4	25.6–33.7
Serum creatinine	95.6	181.5	128.4–140.2	128.3	164.3	142.0–183.0	212.0	134.0	178.0–182.0
eGFR	N/A	34.33	46.11–51.04	50.94	35.2	31.06–34.44	29.1	44.96	31.05–31.89
PH value	N/A	N/A	N/A	N/A		N/A	7.43		N/A
Serum calcium	2.34	2.28	2.16–2.65	2.67	***3.41***	***2.24***–***2.93***	***3.24***	***2.02***	2.20–2.59
Serum phosphate	0.95	0.99	0.65–1.65	1.33	1.29	1.3–1.4	0.94	1.06	1.07–1.41
Urine calcium^+^	5.01	N/A	1.63^b^	N/A	2.51	N/A	2.21	0.29	0.19–0.34
Urine volume (L)	1.50	N/A	2.4	N/A	1.50	N/A	1.00	1.6	1.4–2.0
PTH	5.48	18.05	3.58–5.19	***0.76***	***0.82***	0.79–0.82	0.79	11.44	8.12
25(OH)D	40.61	26.56	36.3–37.2	71.08	44.62	N/A	50.11	27.16	
1,25(OH)_2_D^a^	30.47	165.65	30.93	N/A	N/A	N/A	N/A	N/A	N/A
bALP	8.67	12.94	N/A	N/A	12.02	N/A	14.23	N/A	N/A
CTX	U/A	0.10	N/A	N/A	0.117	N/A	0.154	N/A	N/A

Reference value: 1,25(OH)_2_D 39–193 pmol/L; 25(OH)D 47.7–144 pmol/L; 24-hour urine calcium 2.5–7.5 mmol/24 hours; bALP 11.4–24.6 *μ*g/L; CTX 0.3–0.584 ng/mL; eGFR 56–122 mL/min/1.73 m^2^; PTH 1.6–6.9 pmol/L; serum BUN 3.82–8.86 mmol/L; serum creatinine 53.0–140.0 *μ*mol/L; serum calcium 2.1–2.7 mmol/L; serum phosphate 0.81–1.45 mmol/L. ^a^Measurement of 1,25(OH)_2_D was unavailable since the end of 2013. ^b^24-hour urine calcium was 1.63 mmol when serum calcium was at the level of 2.2 mmol/L.
